# Prediction of disability levels and research on nursing economic costs for elderly people in China

**DOI:** 10.1371/journal.pone.0336605

**Published:** 2025-11-17

**Authors:** Shiyan Lu, Yongxiu Kuang

**Affiliations:** Infectious diseases Department, Wenshan People’s Hospital, Wenshan, Yunnan, China; Korea Institute for Pharmaceutical Policy Affairs, KOREA, REPUBLIC OF

## Abstract

This study aims to solve the problem of the shortage of professional nursing staff and the inability of the quality of nursing services to meet the needs of the elderly. Therefore, a longitudinal research type was adopted for long-term tracking and observation, with a time span from 2017 to 2020. The disability status of the elderly in China was analyzed, and appropriate sample data were selected to construct a comprehensive disability level assessment system. Then, a prediction method for disability scale and level based on the queue element method was proposed. Finally, based on the prediction results, a pension cost optimization strategy was designed, and the current pension methods and economic costs of the elderly were discussed, aiming to providing new ways to solve the deep aging dilemma of Chinese society and families. The results showed that the disability scale prediction method calculated that the growth rate of elderly people from 2025 to 2035 would exceed 65%. Moreover, the prediction error accuracy of elderly people nationwide in 2025 was only −0.03%, and the growth rate of elderly people has reached 69.07%. Compared with mainstream random forests, artificial neural networks, and long short-term memory networks, the research method showed excellent prediction performance, with average absolute error, mean square error, average prediction error, and running time of 0.617576, 0.000053, 0.005007, and 10.24 ms, respectively. The economic cost of nursing for severe disabilities in institutions was nearly 3 times that of mild disabilities and 3.8 times that of home care. The above information shows that the research method can accurately assess the disability level and care needs of the elderly and propose targeted improvement strategies. This method can strengthen the establishment of the nursing service system for disabled elderly people, and build a full chain nursing system including hospitals, elderly care institutions, families, and communities. The study provides strong support for the comprehensive implementation of the long-term care insurance system.

## 1 Introduction

The problem of population aging is accelerating worldwide. Regionally, the elderly population is growing fastest in developing countries, and Asia will become the region with the largest elderly population [1]. North Africa, West Asia, and sub-Saharan Africa, which are currently experiencing a demographic dividend, are expected to experience the fastest growth in the number of older people over the next 30 years [2]. In addition, the map of the world’s “oldest” countries is also changing with social development [3]. Focusing on China’s domestic population, the aging situation is becoming increasingly serious. China’s population aging is characterized by large scale, rapid speed, significant aging trend, prominent problem of getting old before getting rich, urban-rural inversion, and obvious regional differences [4]. At present, the care of the disabled elderly still faces great challenges, the most prominent of which is the huge contradiction between the supply and demand of the elderly population and the high health demand. According to the World Health Organization, the life expectancy at birth in China in 2019 would be 77.4 years, and the healthy life expectancy would be 68.5 years [5]. In other words, the elderly in China have 8–9 years in an unhealthy state, and the problem of unhealthy longevity is more pronounced in the elderly group. By 2024, there would be more than 300 million people aged 60 and above, accounting for about 22% of the total population. The elderly population aged 65 and above would be more than 240 million, accounting for about 16.5% of the total population [6,7].

Many scholars have conducted in-depth discussions and analyses on this issue. The research mainly focuses on two topics: disability assessment and economic costs. In the disability assessment section, N. Sasseville et al. used a cross-sectional approach to document similarities and differences in the forms and consequences of intimate partner violence experiences by persons with disabilities, as well as related risk factors, theoretical explanations, and prevention strategies. Based on this, they developed policies targeting the social determinants of health to prevent intimate partner violence among disabled women [8]. However, this study did not fully consider the influence of cultural background and regional differences on the forms and consequences of violence against women with disabilities, and mainly obtained samples through qualitative methods. In contrast, this study analyzes from both qualitative and quantitative perspectives, focusing on the care needs and economic costs of elderly people with disabilities. S. Shen et al. aimed to analyze the situation of caregivers for disabled individuals, represent the level of care stress, and find effective ways to relieve the stress of this group. The results showed that the care stress of spousal caregivers increased over time, and most were dissatisfied with government care services. Therefore, a comprehensive care system was needed to help reduce the care stress of caregivers [9]. However, this study mainly obtains data through questionnaires, which may have subjective biases, and lacks dynamic analysis of the long-term care process. This study optimizes the care system for Disabled Elderly People (DEP) and conducts a dynamic analysis of changes in long-term care. M. Rome et al. aimed to deeply analyze the elderly care sector in Spain, using a cross-sectional design to evaluate a sample of 353 participants to observe the positive interaction between relationships, task production, and over-commitment. The results showed that over-commitment only had a statistically significant impact on relationship processing-happiness when compared to medium and high levels of over-commitment [10]. However, this study can not establish causal relationships through cross-sectional analysis. In contrast, this study quantitatively analyzes the level of disability and the cost of care, providing support for building a more comprehensive system of care. K. S. Osaki et al. aimed to analyze the relationship between long-term care risks and determinants for the elderly, using data mining methods to analyze the data of elderly people in a certain city in Japan in 2016. The experimental results showed that 16 representative patterns were derived, with age being the most important determinant, followed by motor function, cognitive function, home environment, gender, and place of residence. The relationships between these factors and risk patterns were heterogeneous [11]. This study may have data biases or omissions, while this study constructs a disability level evaluation system and prediction model that can be analyzed qualitatively and quantitatively, providing methodological support for a comprehensive assessment of disability levels. T. Karaca et al. investigated the impact of knowledge and skills on elderly patients with tracheostomy and their caregivers. The study divided caregivers into a control group and an experimental group, using home care and outpatient care models to complete the intervention. The results showed significant differences in the average measurement scores, Zarit Caregiver Burden Scale scores, and average knowledge scores between the two groups [12].

In terms of economic costs, S. Dallmeyer et al. aimed to explore the relationship between participation in sports activities and out-of-pocket medical expenses for the elderly in Europe. The study conducted a cross-national survey on health, aging, and retirement issues in 16 European countries. The results showed that sports activities could be a useful policy tool to reduce the economic burden of out-of-pocket medical expenses for the aging population in Europe [13]. However, this study does not fully consider the impact of the type and intensity of exercise on reducing health economic costs. In contrast, this study focuses on the cost of care for disabled older adults and improvement strategies aimed at reducing the economic cost of long-term care. R. Hussain et al. found that the person-centered legislative goals of the aged care and services sector in Australia have been limited in their translation into practice. Therefore, they conducted qualitative in-depth interviews with stakeholders. The research results indicated that a higher-quality support system could be ensured by improving workforce planning strategies, enhancing the skills of existing staff, and including location-based collaboration, among other measures [14]. The sample sizes of the above studies are relatively small, and this study uses a large dataset to conduct evaluations from both a qualitative and quantitative perspective, providing a scientific basis for improvement strategies.

In summary, the research results mainly focus on the influencing factors and economic costs of evaluating DEP. However, these methods cannot comprehensively evaluate the current disability status of the elderly, and the dimensional analysis for prediction is relatively simple. The research objective is to integrate multiple dimensions to comprehensively evaluate the disability status of elderly people, providing a basis for subsequent care needs and cost analysis. Moreover, in response to the dynamic and uncertain nature of aging and disability issues, a prediction method that can handle time series data and transition probabilities is proposed. In addition, a more scientific quantitative analysis method should be developed to provide a basis for optimizing the allocation of care resources. Therefore, based on the obtained data, a Comprehensive Disability Assessment system for the Elderly (CDAE) is constructed. Then, a prediction method for the Scale of Elderly Disability (SoED) is constructed by combining the Queue Elements method (QE) and the Markov chain. Finally, the care methods and related economic costs are analyzed.

The innovation of the research is mainly in the following three points. The first point is to propose a joint QE method and the Markov chain model to predict the scale of DEP in various dimensions, thus providing solid data analysis support for proposing targeted improvement strategies in the future. The second point is to construct a cost accounting model for the care of disabled elderly, which quantifies the economic costs of different care methods and different dimensions of disability levels. The third point is to explore in depth the economic incentive mechanism for long-term care of DEP and propose sustainable economic support plans.

## 2 Methods

### 2.1 Sample data analysis and processing

This study is based on the CHARLS database and publicly available official data, such as the China Statistical Yearbook and the China Civil Affairs Statistical Yearbook, for secondary analysis. The CHARLS project has been approved by the Ethics Review Committee of Peking University during the raw data collection phase, and all participants in the survey have signed informed consent forms. The data used in this study are anonymized public data, which do not involve personal privacy leakage risks and comply with scientific research ethical norms.

At present, China’s aging population is becoming increasingly significant, and the degree of aging is increasing year by year. In 2023, the number of people over 60 years old in China will reach 297 million, accounting for 21.1% of the total population, while the number of people over 65 years old will exceed 217 million, accounting for 15.4% [15,16]. As they age, the functions of the elderly will continue to decline, and due to diseases, accidents, etc., many elderly people lose their ability to look after themselves [17]. Given the survey data, the current amount of DEP in China is about 35 million. According to estimates, by 2035, this number will reach 46 million. Among them, providing care for disabled elderly is a fundamental need for Elderly Care Services (ECSs) and also a top priority for many people who are anxious about elderly care. Cracking the problem of caring for DEP and addressing the demand for ultra-short scale nursing services are crucial to the overall development of the country and the well-being of millions of people [18,19]. Therefore, this experiment adopts a longitudinal research type, through long-term tracking and observation, to analyze the changing trend of disability levels among Chinese elderly people and their economic costs of care. The research period is from 2017 to 2020, and the data are mainly from the China Health and Retirement Longitudinal Study (CHARLS) database. Moreover, for subsequent analysis of the scale of disability prediction, the research also introduces the *2023 China Statistical Yearbook, China Civil Affairs Statistical Yearbook, China Urban and Rural Elderly Living Conditions Sample Survey, Social Service Development Statistical Bulletin, and the Seventh National Population Census Bulletin* [20–22]. Among them, the CHARLS dataset covers 150 county-level units, 450 village-level units, and about 10,000 households with 18,000 individuals. The dataset is designed with a wide range of data, including basic personal information, health status, physical measurements, work, income, assets, family structure and economic support, medical service utilization and medical insurance, consumption, retirement and pension, and basic community conditions, etc. [23,24]. In terms of population and sampling, the target group is selected as Chinese elderly people aged 60 and above, with a sample size of 2,967 valid questionnaires. They are all from respondents aged 60 and above in the CHARLS database. The inclusion criteria are as follows: ① Age ≥ 60 years old. ② Exclude samples with a large number of missing or abnormal data. ③ Have complete health status, physical measurements, and functional assessment data. ④ Have data from the Mini-Mental State Examination. The selection process is as follows: First, suitable respondents are screened from the CHARLS database, totaling approximately 18,000 individuals, and then the obtained samples are processed. Ultimately, 2,967 valid samples are determined, covering different genders, urban and rural backgrounds, and age groups, ensuring the representativeness and diversity of the samples.

The specific sample data processing process first obtains basic personal information through the CHARLS dataset. The health and functional module obtains self-assessment of health, participation in physical and social activities, and illness. The family module selects the relevant personnel and time consumption for nursing in the home. The cognitive and depression module collects data from a simple mental state examination. The variables measured are basic personal information, health status, physical function and self-care ability, care needs and services, economic costs, and social supports and services. Abnormal data present in the sample are directly deleted. Vague data such as “not evaluated’‘ and “unclear’‘ have also been removed. Missing data in the sample can be subjected to multiple imputation or deletion based on the reasons for the missing data and the current situation [25,26]. After the above data processing operations are completed, merge conversion can be used to process the data that meets the requirements and assign values at the same time. Table 1 shows the specific content.

**Table 1:** Different types of data assignment representation.

Type	Project	Option	Score
Health and disease status	Self assessed health status	Very bad	0
		Not good	1
		Commonly	2
		Good	3
		Very nice	4
	Disease status (including chronic disease, hypertension, diabetes, arthritis/rheumatoid disease and digestive system disease)	Be ill	0
		No illness	1
		For each additional category of disease, 1 point will be deducted	/
Participate in physical and social activities	Physical activity (3 categories in total)	Participate in an activity	1
	Social activities (11 categories in total)	Participate in an activity	1
Physical functional status	Physical function and self-care ability (There are a total of 11 questions. If there are no difficulties in choosing questions 1–9, you can skip questions 10–15, and the score for filling in the number of times is set to 1 point.)	Unable to complete	0
		Having difficulties and needing help	
		Difficult to complete	
		Nothing to	1
Cognitive ability status	Simplified Mental Health Examination Scale (5 questions in total)	Correct answer	1
		Incorrect answer	0
	Self evaluation of memory status	Not good	1
		Commonly	2
		Good	3
		Very good	4
		Good factory	5

**Fig 1:**
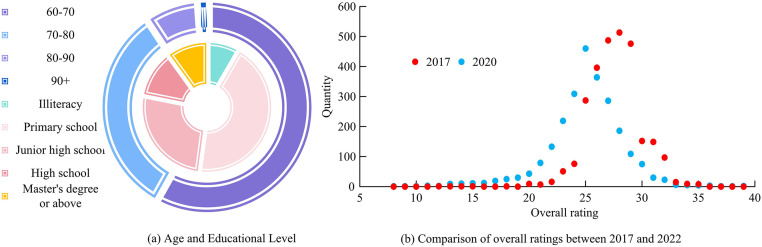
Distribution of sample age and education level, and changes in the total score of comprehensive evaluation in 2017 and 2020.

Table 1 contains four types of condition assessments, each with specific options and corresponding scores for each item. By evaluating and assigning values based on the above situation, the required valid data can be obtained. The scores from different dimensions and the total score data are shown in Fig 1.

Fig 1(a) shows the ratio of age to education level. At present, the proportion of people aged 60–70 is 59.3%, while the proportion of people aged over 80 is only about 8%. This indicates that China’s elderly are concentrated in the younger age group. Due to the limitations of the times, only a small number of elderly people currently receive higher education, while the vast majority of elderly people receive only junior high school or lower education. Fig 1(b) shows the comprehensive assessment data, and overall, the assessment in 2020 is significantly lower than that in 2017. This indicates that as the elderly continue to age, their participation in activities, physical health functions, etc., will become weaker, corresponding to more severe disabilities.

### 2.2 CDAE and scale prediction methods

**Fig 2:**
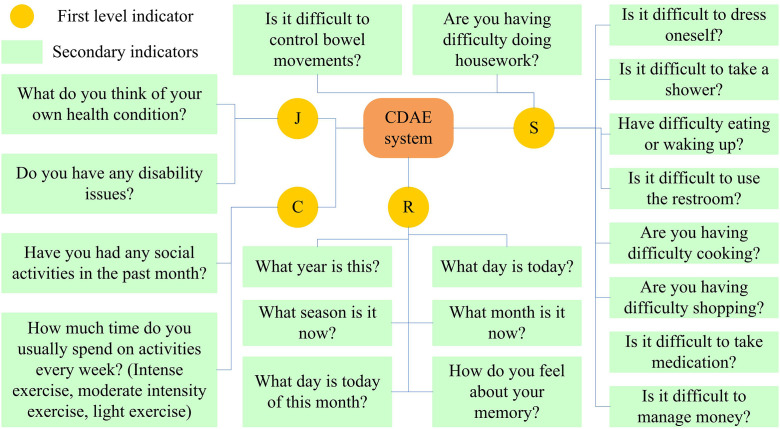
CDAE system diagram.

After the above data processing and analysis are completed, a CDAE system can be constructed based on the extracted four types, including four dimensions: physical function (S), health disease (J), participation in activities (C), and cognitive ability (R). The specific content is shown in Fig 2.

**Fig 3:**
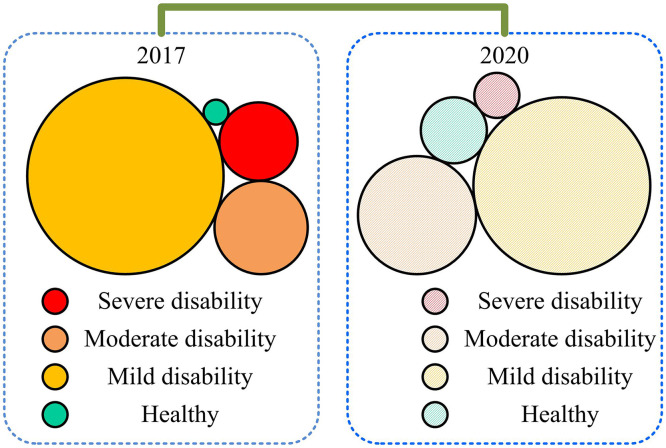
Distribution of disability levels among elderly people in 2017 and 2020.

In Fig 2, there are a total of 4 primary indicators and 20 secondary indicators. Through this evaluation system, a comprehensive assessment of the disability situation of the elderly can be conducted. The data show that due to the fact that the body is the foundation of social participation, these two dimensions have a high proportion, while the other two dimensions have a relatively small weight allocation due to their strong subjectivity. Therefore, the weights of S, J, C, and R are 0.2513, 0.3201, 0.1968, and 0.2318, respectively. The total score corresponding to the sample can be obtained through calculation. Assuming that the grading of the sample approximately follows a normal distribution. As an evaluation criterion, the following evaluation methods can be obtained. The group distance is set at 5 points, and elderly people who do not exceed 20 points are grouped together, which is the severe disability level. A score of 21–25 can be classified as a moderate disability level. Through the score of different dimensions, the weight of the development can be further calculated. Therefore, it can obtain the proportion of different dimensions in the grade evaluation, and thus realize the verification of the disability level of the elderly. Subsequent levels are classified as mild-disability level and health level, etc. In this way, the distribution maps of disability levels among the elderly in 2017 and 2020 can be obtained, as shown in Fig 3.

Fig 3 shows that compared to 2017, the quantity of elderly with mild disability and health levels has been continuously decreasing in 2020, and the proportion of people with health levels is less than 10%. This means that the current care issues related to disabled elderly continue to receive attention from the government and society.

After the construction and improvement of the CDAE system, further design of SoED prediction methods can be carried out. Markov chain is a powerful mathematical tool that can model and predict time series data in various fields, including finance. Moreover, it has flexibility and good predictive performance, can provide solid mathematical principles, and can also deeply understand system behavior and provide information for decision-making [27–29]. Therefore, this study uses it as the basic model for SoED prediction. Specific operation: First, assuming that the number of DEP in a certain year is α , which belongs to a random variable. If analyzing the changes in the number of people at regular intervals, it is necessary to use time interval t to represent α(t). The corresponding discrete space states are represented using four levels of elderly disability, with moderate disability being replaced by 1–4 levels from healthy to final, and different states being interconnected. By following a discrete-time Markov chain for the state changes from a to a+1 years old, the corresponding state space can be obtained as A(t). Conditional probability expression is shown in equation (1).


$$P_{r}\left\{A(b+t)=j|\;A(b)=i,A(u)=A(u),0\leq u<b\right\}=P_{r}\left\{A(b+t)=j|\;A(b)=i\right\}$$ (1)

In equation (1), b is any time. i and j are both disability levels for any elderly person. By substituting the disability data into equation (1), the corresponding disability level transition probability $P_{z}$ can be obtained, as shown in equation (2).


$$P_{z}=\left[\begin{array}{c} P_{1,1}(2017,2017+t)\;P_{1,2}(2017,2017+t)\;P_{1,3}(2017,2017+t)\;P_{1,4}(2017,2017+t)\\ P_{2,1}(2017,2017+t)\;P_{2,2}(2017,2017+t)\;P_{2,3}(2017,2017+t)\;P_{2,4}(2017,2017+t)\\ P_{3,1}(2017,2017+t)\;P_{3,2}(2017,2017+t)\;P_{3,3}(2017,2017+t)\;P_{3,4}(2017,2017+t)\\ P_{4,1}(2017,2017+t)\;P_{4,2}(2017,2017+t)\;P_{4,3}(2017,2017+t)\;P_{4,4}(2017,2017+t) \end{array}\right]$$ (2)

In equation (2), 1–4 correspond to the disabled state, respectively. To accurately predict the number of disability levels among elderly people each year, it is necessary to calculate a transition probability matrix with t = 1 based on the known transition probability matrix. However, there is a significant difference between the transition matrix calculated multiple times and the 3-year period $P_{z}$ obtained directly, so further correction is needed to achieve an error within 5%. By calculating the number of disability levels for each elderly person from 2017 to 2020, the corresponding transition state probability matrix $P_{z-3}$ can be obtained, as expressed in equation (3).


$$P_{z-3}=\left[\begin{array}{c} 0.3382\;0.5819\;0.0798\;0.0037\\ 0.0602\;0.6669\;0.2515\;0.0250\\ 0.0198\;0.3869\;0.4876\;0.1057\\ 0.0000\;0.1100\;0.2400\;0.6500 \end{array}\right]$$ (3)

Equation (3) indicates that elderly people who belong to the health level have a 0.37% probability of becoming severely disabled and a 58.19% probability of becoming mildly disabled after 3 years. Moreover, elderly people with mild disabilities have the highest probability of maintaining their original state after 3 years, at 66.69%. The probability of elderly people with severe disabilities still maintaining their original state reaches 65%. To further predict the probability of disability each year, it is necessary to divide the disability level within 3 years into three parts. It is assumed that the disability level changes with the same probability every year, the relationship between the transition probability matrix $P_{3}$ with t being 3 and the transition probability matrix with t being 1 is as follows $P_{1}$, $P_{3}=P_{1}^{3}$. Therefore, $P_{3}$ can be treated as open. However, in practical applications, matrix square root cannot be processed, so this study introduces the Step Size Transformation of Transfer Matrix (SSTTM) method for processing. The matrix $P_{3}$ is represented by equation (4).


$$P_{3}=\left[\begin{array}{c} G_{11}\;G_{12}\;G_{13}\;G_{14}\\ G_{21}\;G_{22}\;G_{23}\;G_{24}\\ G_{31}\;G_{32}\;G_{33}\;G_{34}\\ G_{41}\;G_{42}\;G_{43}\;G_{44} \end{array}\right]$$ (4)

In equation (4), $G_{ij}$ is an element of the matrix. Except for the element $G_{ii}$ of $P_{3}$, all other elements are divided by 3 for processing, resulting in the formula for $P_{1}$, as shown in equation (5).


$$P_{1}=\left[\begin{array}{c} g_{11}\;g_{12}\;g_{13}\;g_{14}\\ g_{21}\;g_{22}\;g_{23}\;g_{24}\\ g_{31}\;g_{32}\;g_{33}\;g_{34}\\ g_{41}\;g_{42}\;g_{43}\;g_{44} \end{array}\right]$$ (5)

In equation (5), $g_{ij}$ is an element of $P_{1}$. Next, the modified matrix $P^{\prime }$ is expressed as equation (6).


$$P^{\prime }=\left[\begin{array}{c} {}*20c1-\frac{\left(A_{12}+A_{13}+A_{14}\right)}{\text{3}}\frac{A_{12}}{\text{3}}\frac{A_{13}}{\text{3}}\frac{A_{14}}{\text{3}}\\ \frac{A_{21}}{\text{3}}1-\frac{\left(A_{21}+A_{23}+A_{44}\right)}{\text{3}}\frac{A_{23}}{\text{3}}\frac{A_{24}}{\text{3}}\\ \frac{A_{31}}{\text{3}}\frac{A_{32}}{\text{3}}1-\frac{\left(A_{31}+A_{32}+A_{34}\right)}{\text{3}}\frac{A_{34}}{\text{3}}\\ \frac{A_{41}}{\text{3}}\frac{A_{42}}{\text{3}}\frac{A_{43}}{\text{3}}1-\frac{\left(A_{41}+A_{42}+A_{43}\right)}{\text{3}} \end{array}\right]$$ (6)

The probability matrix of disability transition in elderly people over a period of one year can be obtained through equation (6). However, there is still a significant error at this time, so further adjustment is needed through equation (7).


$$e=\left|\frac{a_{act}-a_{est}}{a_{act}}\right|$$ (7)

In equation (7), e is the correction error. $a_{act}$ and $a_{est}$ are the actual and predicted values. The modified transition probability matrix expression $P_{1}^{\prime }$ for $P_{1}$ is shown in equation (8).


$$P_{1}^{\prime }=\left[\begin{array}{c} 0.4830\;0.4129\;0.0944\;0.0097\\ 0.0347\;0.7359\;0.1989\;0.0305\\ 0.0185\;0.2760\;0.6233\;0.0822\\ 0.0028\;0.1079\;0.1869\;0.7024 \end{array}\right]$$ (8)

$P_{3}$ can be directly calculated to represent the actual number of disabled states in 2020. The $P_{1}$ after square root represents the predicted number of disabled states in 2020 through step size conversion, and finally the correction error is obtained through equation (7). To further understand the above method, the study illustrates the operational mechanism of the research method through simplified practical cases. Assuming there are 1,000 healthy elderly people in a certain area, based on historical data, the probability of each healthy elderly person maintaining health within one year is 90%, the probability of mild disability is 9%, and the probability of death is 1%. According to the QE method, it is predicted that 50 new individuals over the age of 60 will join the health queue in the next year. The number of healthy elderly people in the next year can be calculated as: 1000 × 90% + 50 = 950 people. At the same time, 90 elderly people with mild disabilities are newly generated, accounting for 1000 × 9%. On this basis, by continuing to apply corresponding state transition probabilities to these 90 mild DEPs, such as 80% maintaining the status quo, 15% transitioning to moderate disability, and 5% passing away, recursive predictions of the population size of each disability level in the next few years can be achieved. This method of combining population base prediction with state transition simulation takes into account the dynamic changes in the age structure of the population and captures the stochastic transition characteristics of individual health states. This method provides a scientific basis for formulating long-term policies to deal with population aging.

### 2.3 Elderly care methods and economic costs

Accompanying the disability of the elderly is the issue of disability care. The current main nursing methods include home care, institutional care, and community care. The most common is family care, which is due to the constraints of China’s social development degree and economy, as well as the influence of traditional culture and moral concepts [30,31]. Family care refers to elderly people spending their later years at home and receiving home services. The advantage of this mode lies in its ease of movement and comfort, without the need to adapt to a new environment, while also enjoying professional care services. Institutional care refers to living in specialized elderly care facilities, including nursing homes, senior apartments, etc. The advantage of this model lies in its specialization and scale, and it can provide comprehensive medical and nursing services, but it lacks the warmth of a family. Community care is a model between institutional care and home care, which involves setting up small or micro nursing homes within mature communities to provide centralized care services for the elderly. It combines the professionalism of institutional care with the warmth of family care, providing a convenient and comfortable environment for the elderly. Previous statistical data showed that in 2021, 80% of the elderly in China reported suffering from chronic diseases, with little difference between urban and rural areas. The proportion of males and females is 48.3% and 51.7%. The population aged 60–69 accounts for 56.2%, and the proportion of elderly over 80 years old is 13.4%. In terms of health and medical conditions, the total proportion of self-rated health status as very good or relatively good is 42.7%. For the status of care and nursing services, 88.4% of the elderly are able to take care of themselves, while 13.2% of them need to be taken care of in their daily lives. The proportion of people willing to receive care services in elderly care institutions is 7.7%. The top five types of community (village) ECSs with the highest proportion of elderly demand are home medical services, meal assistance services, cultural and entertainment services, health education services, and home chores services. In terms of economic situation, the annual per capita income of elderly people in China was 32027.4 yuan, and 22.8% of elderly people have been hospitalized in 2020, with an average of 9620 yuan for hospitalization medical expenses paid by individuals. In terms of social participation, 26.1% of the elderly participated in various social groups or organizations, such as elderly mutual aid organizations, cultural and recreational organizations, etc. For the protection of rights and interests, over 90% of the stable proportion believed that their legitimate rights and interests have been properly protected. In terms of spiritual and cultural life status, 49.3% of people participated in various daily leisure activities.

The economic cost of long-term care for DEP can be calculated using equation (9).


C=d×T×ζ×ψ (9)

In equation (9), C, d, and T correspond to the nursing cost, monthly nursing days, and daily nursing hours of a disabled elderly person. ζ and ψ are the nursing cost coefficients corresponding to the disability level and the time value of nursing staff. The data in equation (9) are all from the 2020 CHARLS dataset. ζ is the coefficient for selecting the labor cost of family members, the average hourly wage of on duty elderly care workers, and the disability level of individual institutions. Table 2 shows the economic costs of different care methods for DEP.

**Table 2:** Comparison of monthly economic costs between institutional care and home care models for elderly people with different levels of disability.

Nursing methods	Institutional nursing			Home care
	Mild disability	Moderate disability	Severe disability	
C /USD	518.95	629.47	1389.39	397.71
d /day	20.94	21.95	24.42	21.63
T /hour	4.46	4.84	8.58	4.83
ζ	1.81	1.93	2.16	1.24
ψ	3.07			

In Table 2, the economic cost of institutional care is greatly higher than that of home care, making it still the preferred nursing method for the elderly. As the level of disability continues to increase, the corresponding economic costs are also constantly rising. Among them, the economic cost of nursing for severe disabilities in institutions is nearly three times that of mild disabilities and 3.8 times that of home care. Therefore, the future annual nursing economic costs can be calculated by combining the disability scale prediction method, providing solid data support for subsequent improvement measures. The final model assumptions are as follows: First, the transition of the disabled state is random, and the transition probability matrix can be estimated using historical data and corrected for errors using correction methods. Second, the growth trend of care costs is consistent with the growth trend of the disabled elderly population, and the prediction of future care costs is based on linear or nonlinear extrapolation of existing data. Third, the allocation of resources between home care and institutional care has remained relatively stable during the study period and will not undergo significant adjustments due to policy changes or economic fluctuations. Fourth, the error of the forecasting model is within an acceptable range of 5%.

## 3 Result

### 3.1 Assessment results of disability levels for elderly in China

**Fig 4:**
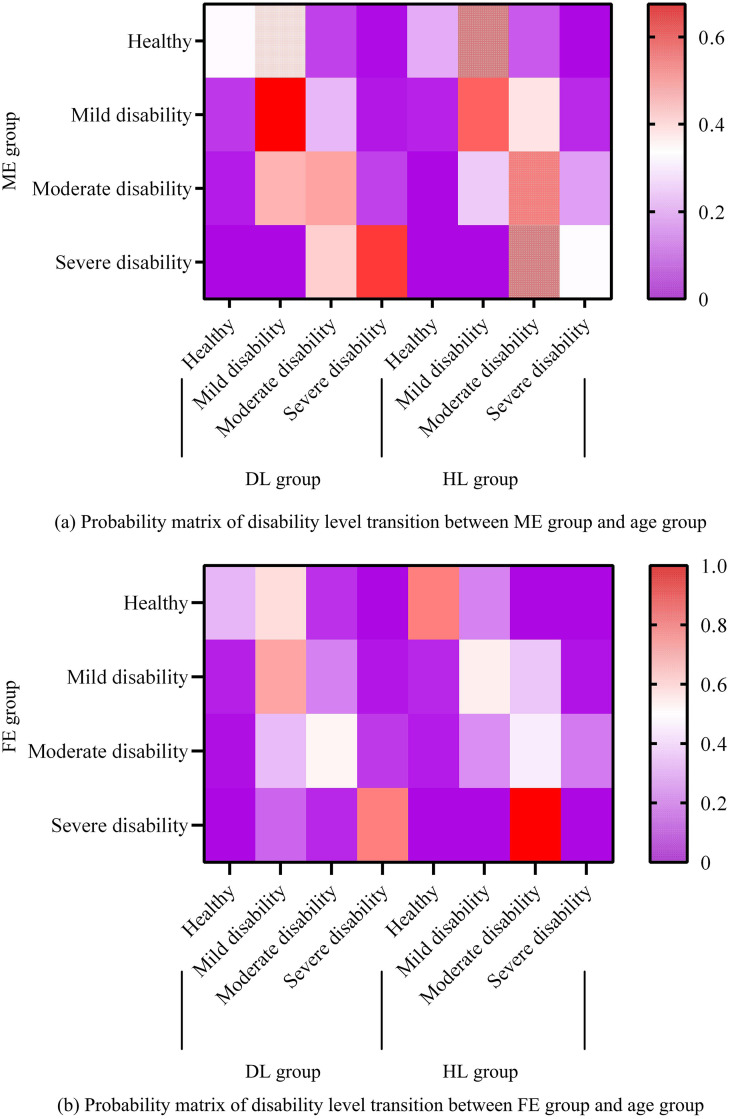
Results of disability level transition probability matrix based on 3-year age and gender groups.

This study first analyzes the differences in disability levels among different types of elderly people using the SSTTM method. The first method is to divide the research subjects into the younger group (DL) under 80 years old and the older group (HL) over 80 years old based on age. The second type is divided into urban group (TN) and rural group (RL), male group (ME) and female group (FE) based on gender and urban-rural affiliation. This study first explores the disability levels of age and gender groups based on a 3-year period, and calculates the corresponding transition probability matrix, as displayed in Fig 4.

**Fig 5:**
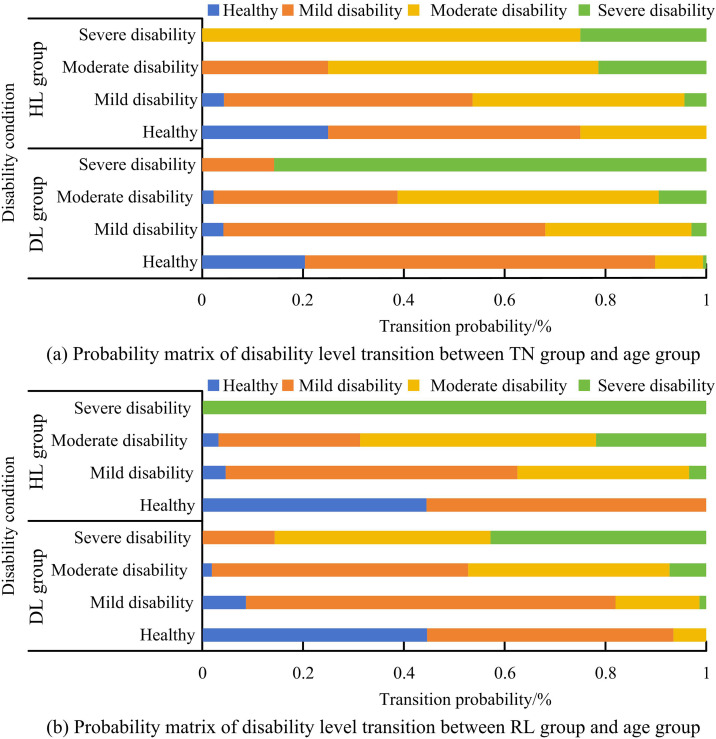
Results of the disability level transition probability matrix for urban rural and age divided groups based on a 3-year period.

Fig 4 shows the changes in disability levels among different age groups in the ME and FE groups. In Fig 4 (a), there is no significant difference in physical fitness among the elderly in the DL group, and the transition probabilities corresponding to different disability levels are relatively small, with a higher probability of maintaining mild and severe disability levels. In Fig 4 (b), the changes in the DL group are consistent with those in the ME group, but the HL group has the highest probability of maintaining a healthy level, at 77%. This indicates that older women have a lower probability of entering the mild disability level, and their aging rate is slower than that of men, with less fluctuation between different levels. There is no difference between genders in the DL group, but without a discrepancy in the HL group. Fig 5 shows the results of the disability level transition probability matrix based on a 3-year period of urban-rural and age-divided groups.

**Fig 6:**
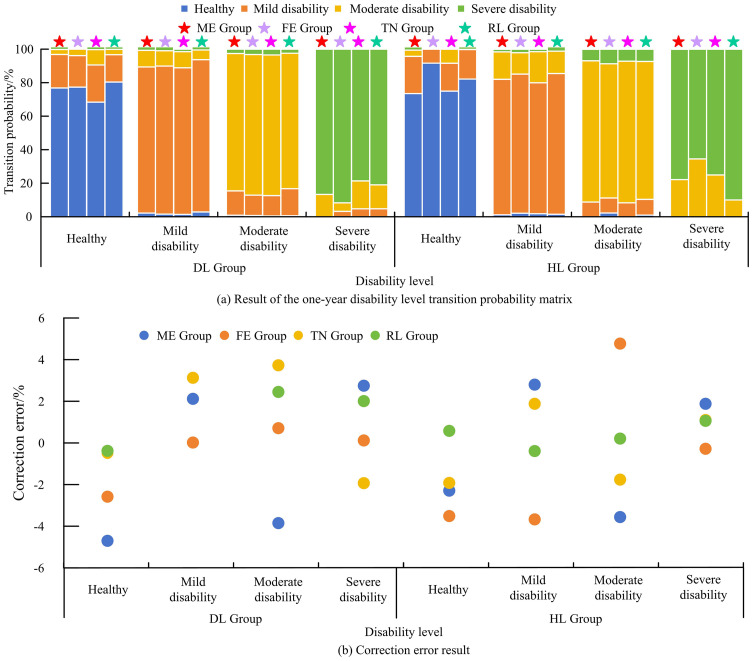
The results of the one-year disability level transition probability matrix and the correction error results.

Figs 5 (a) and (b) show the transition probabilities of disability levels for different age groups in the TN and RL groups. There are differences between the TN and the RL in different age groups, with the DL having significantly better physical fitness than the TN group. This may be because the elderly in the RL group engage in field work year-round, which not only maintains their health but also promotes their happy mood. In addition, the countryside has beautiful scenery and rich natural resources, relatively good air quality, and closer communication and interaction among people. After square root processing, the one-year disability level transition probability matrix and error correction results can be obtained, as shown in Fig 6.

Fig 6 (a) shows the result of the disability level transition probability matrix. In the state transitions of severe disability levels in different classification groups, there are still many data transition probabilities of 0. The probability of transitioning from disability level to health level is 0, which is consistent with the actual situation. In addition, compared to the results of the 3-year matrix, the diagonal elements of the matrix are significantly increased. This indicates that the physical condition of elderly people with different levels of disability is relatively stable within one year, generally maintained at 70% −90% of their original state. Fig 6(b) shows the result of the error correction. It can be observed that the minimum and maximum correction errors in each group are 0.03% and 4.72%, respectively, which are within the set error range. This indicates that the SSTTM method used is effective, feasible, and accurate, and can provide strong data support for predicting the scale of disability in the future.

### 2.2 Prediction results of disability among elderly in China

To more accurately predict the changes in the scale of disability among elderly people in China, this study combines the QE method to complete the prediction. The QE method mainly considers the fertility, mortality, and migration of the population comprehensively, and for the elderly, only death needs to be considered. Therefore, this study uses mortality rates of different age groups living separately and by genders for subsequent prediction. First, to evaluate the effect of the proposed prediction model more scientifically, the study uses other existing mainstream methods to conduct comparative experiments, namely Random Forest (RF) and Long Short-Term Memory Network (LSTM), and Artificial Neural Network (ANN). In addition, the current mainstream Mean Absolute Error (MAE), Mean Square Error (MSE) and Average Prediction Error (APE), and Running Time (RT) are selected as evaluation indicators. Among them, the predicted value is the result calculated based on the microscopic data and the prediction result obtained by the above machine learning, and the measured value is the seventh national population census data. The performance results of different forecasting methods can be compared, as shown in Table 3.

**Table 3:** Comparison of performance results of different prediction methods.

Research method	MAE	MSE	APE	RT/ms
RF	0.611537	0.000058	0.004982	98.27
LSTM	0.61999	0.000052	0.004942	75.23
ANN	0.547126	0.000032	0.002769	63.27
Research method	0.617576	0.000053	0.005007	10.24

**Fig 7:**
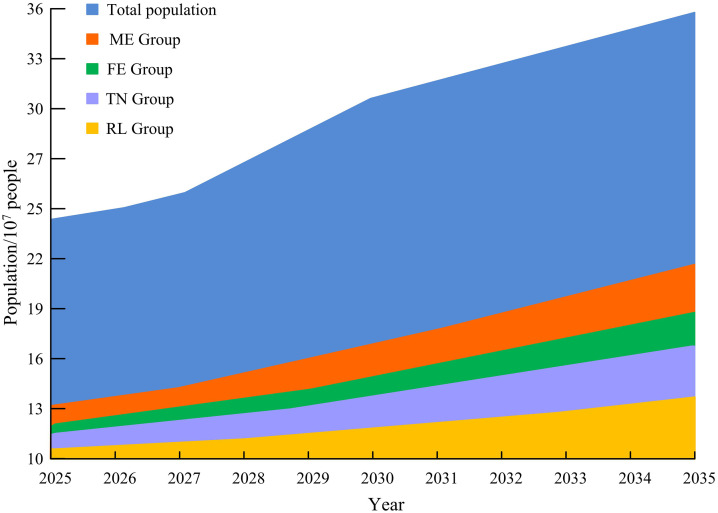
Prediction results of the number of elderly people in the DL group.

According to Table 3, the prediction accuracy, error control, and prediction efficiency of the research method are excellent, and the MAE, MSE, APE, and RT are 0.617576, 0.000053, 0.005007, and 10.24, respectively. Although the prediction performance of the research method is slightly lower than that of ANN to some extent, its RT performance is the best, with an improvement of 518%. Fig 7 shows the predicted number of elderly individuals in the DL group.

**Fig 8:**
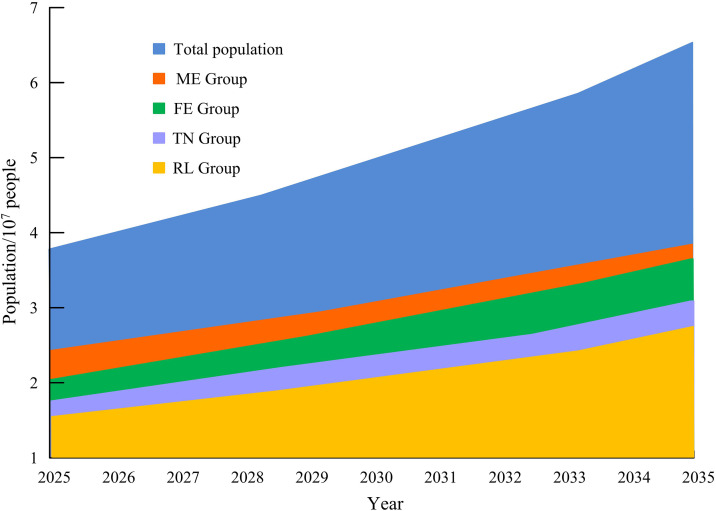
Prediction results of the amount of elderly in the HL group.

In Fig 7, different groups show a growth trend, and the population growth of the ME group is lower than that of the FE group, while the population growth of the RL group is the slowest. By 2035, the total population growth rate of the DL group will reach 38.26%, while the population growth rate of the TN group will exceed 65%. This indicates that the nursing and physical therapy needs of the elderly are rapidly increasing in the future, and this issue urgently needs to be addressed. Fig 8 predicts the number of elderly people in the HL group.

**Fig 9:**
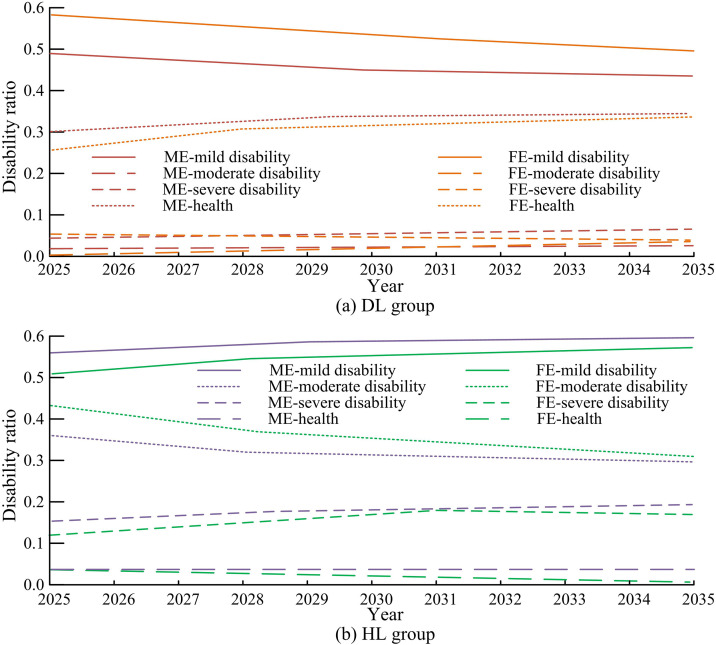
Prediction results of the proportion of elderly disability scale.

In Fig 8, overall, all groups show a growth trend, but their growth base is lower compared to the RL group. In 2035, the overall growth rate will reach 69.07%, with a growth rate of 73.26% for the TN group. By combining the prediction of the number of different categories of groups and the proportion of disability levels mentioned above, the predicted proportion of SoED can be obtained, as shown in Fig 9.

**Fig 10:**
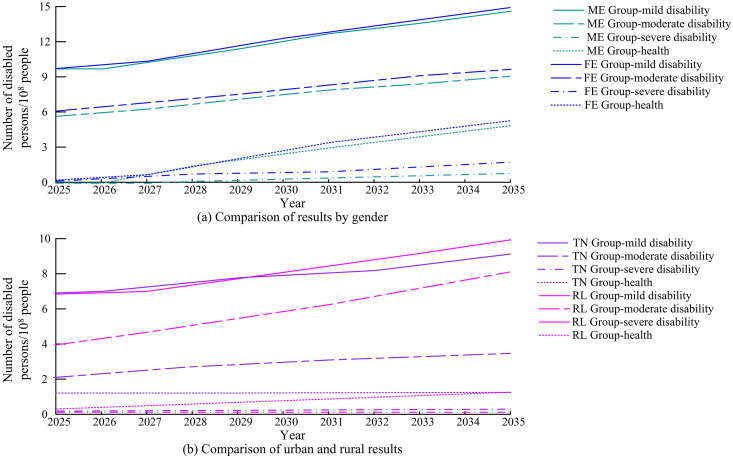
Prediction results of disability scale for DL group.

Figs 9(a) and 9(b) correspond to the predicted disability scale proportions between the DL and HL groups. It can be concluded that the gender differences are not significant in the DL group, but vary greatly in the HL group. The proportion of women’s health is much higher than that of men, and with the continuous increase in years, the advantage of women’s life expectancy continues to emerge. By calculation, it can be concluded that the comparison of health levels between men and women has significant statistical significance (*p* < 0.05). By multiplying the predicted quantity by the corresponding disability level prediction ratio, the corresponding disability scale prediction result can be obtained. The predicted final disability scale of the DL group is shown in Fig 10.

**Fig 11:**
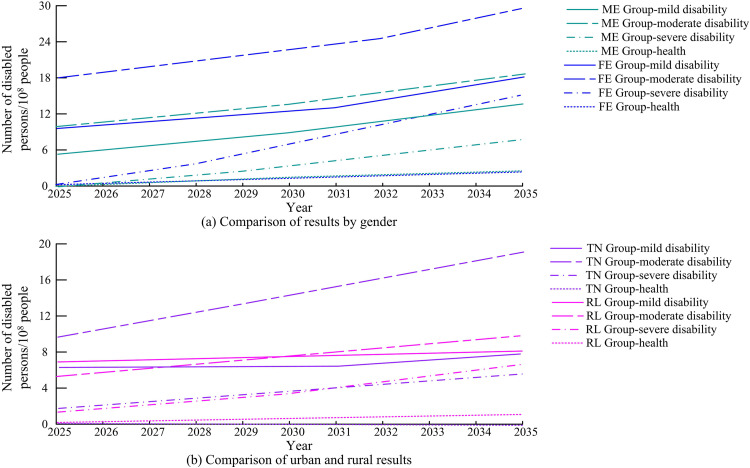
Prediction results of disability scale for HL group.

Figs 10 (a) and 10 (b) show the predicted disability levels for gender and urban-rural populations. In the prediction results of disability scale for different groups, the degree of aging is constantly deepening, and the number of DEP is also rapidly increasing. The number and proportion of elderly people with mild disability in the RL group are higher than those in other groups, while the number of people with severe disability in the FE group is significantly higher than that in the ME group. Fig 11 shows the predicted distribution of the final disability scale for the HL group.

Figs 11 (a) and 11 (b) show the predicted disability scale based on gender and place of residence in the HL. The distribution of each group category in the HL is relatively scattered, and gender differences are very obvious. In addition, the health level of the ME group is almost not displayed, while the health level of the FE shows significant performance and a continuous increasing trend with the increase of years. In 2035, the number of moderate disability levels will rapidly increase, doubling compared to 2025. In summary, although the prediction results of this study are based on a rigorous QE method and the Markov chain model, there is a certain degree of uncertainty in population health prediction itself. To reflect this uncertainty, the study further provides an analysis of the prediction range under different scenarios. Beyond the baseline scenario, the study sets two scenarios: optimistic and pessimistic. The optimistic scenario assumes that medical technology progress and health promotion policies are significantly effective, and the probability of disability transfer improves by 2% annually. In the pessimistic scenario, the rising incidence rate of chronic diseases and the shortage of nursing resources are considered, and the probability of disability transfer worsens by 2% every year. Analysis shows that by 2035, the predicted size of the disabled elderly population in China may decrease by about 8% compared to the baseline under an optimistic scenario, while it may increase by about 11% under a pessimistic scenario. This interval estimation helps decision-makers understand the possible range of fluctuations in the predicted results.

### 3.3 The economic cost and improvement measures of disability care for elderly in China

**Fig 12:**
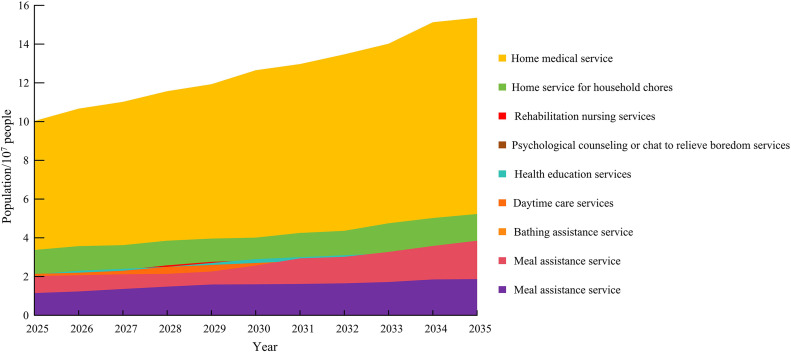
Prediction of the Demand Scale for Major Community ECSs for Chinese Elderly by 2035.

According to the data from the fourth (2015) and fifth (2021) “Statistical Bulletin on the Sampling Survey of the Living Conditions of Elderly People in Urban and Rural China and the Development of Social Services”, it can be assumed that the proportion of long-term care needs of the elderly will remain unchanged in the future. Furthermore, based on the predicted disability scale mentioned above, the future demand for elderly services is further predicted, as shown in Fig 12.

**Fig 13:**
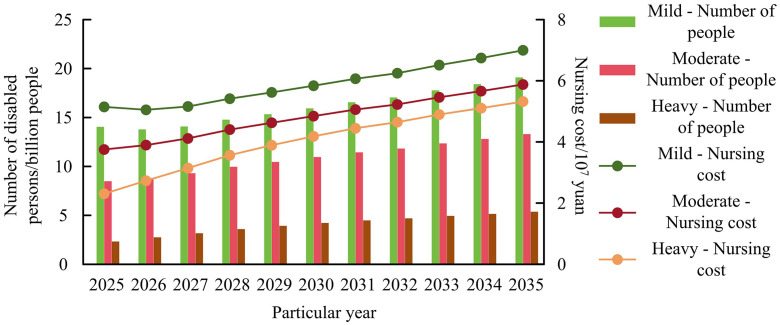
Prediction of total long-term care costs for elderly people in China from 2025 to 2035 based on the distribution of disability levels.

In Fig 12, the demand for home medical services among the elderly will be the highest in the future, reaching approximately 1.5 billion people by 2035. This is due to the worsening aging population, weak primary healthcare service capabilities, inadequate hardware environment, and inadequate medical standards. Fig 13 shows the results of predicting the total cost of long-term care for the elderly by combining the economic costs of nursing for various disability levels.

Fig 13 shows that with the increase in years, the economic cost of long-term care for the elderly is also growing rapidly. This requires the government to provide corresponding subsidies to alleviate the economic cost of caring for the elderly. The cost of nursing at the severe level will reach its highest value in 2035, approximately 7.2*10^7^ yuan, while the cost of nursing at other levels will be within 6*10^7^ yuan.

## 4 Discussion

The experimental results showed that compared with 2020, the number of seniors with mild disability and healthy grade showed a decreasing trend, while the number of seniors with severe disability grade increased. Moreover, by 2035, the cost of care for severe disability grades was expected to reach about 7.2 × 10^7^, which was a heavy burden on families and society. Most of the elderly were more inclined to home care, but the pressure on home care workers was greater, and the satisfaction of the care services provided by the government was low. As an intermediate model, community care combined the warmth of home care with the professionalism of institutional care, but its development was lagging behind. The analysis section delineated the formulation of short-term and long-term implementation plans. In the short term, relevant policies could be introduced in 2026 to support the construction of embedded community ECS institutions, while expanding the construction of community ECS facilities to ensure that each community had at least one ECS point. By the year 2027, the three-tier ECS network, comprising counties, towns, and villages, would undergo enhancements to ensure that the service coverage rate exceeds 80%. Additionally, the visiting care service mechanism for individuals experiencing social isolation, those with no family support, and elderly individuals would be augmented to ensure that the service coverage rate reaches over 70%. In the long run, ECS legislation can be fully implemented within the next ten years to ensure the standardization and standardization of ECS and achieve full coverage of the ECS network. It can also ensure that every elder can receive timely and effective care services, establish an incentive mechanism for ECS, and attract more professionals to participate in ECS. As indicated by the established hierarchy of resource allocation, policies and regulations are of the utmost importance. The formulation and implementation of legislation on ECSs, as well as the assurance of legal framework and policy support for disabled ECSs, constitute the primary task at hand. The next focus is on the optimization of the service system, including deepening the institutional reform of public elderly care institutions, increasing the supply of inclusive care services, and the ultimate goal of talent training and team building, thereby enhancing the incentive mechanism for ECS talents.

The physical fitness of the rural elderly was significantly better than that of the urban elderly, and they showed a lower risk of disability in both the younger and older age groups. The above results might be due to the fact that the rural elderly had to work in agriculture, which, to some extent, exercises their bodies and makes them healthier. Moreover, data from CHARLS showed that the elderly participate more in agricultural work, and the marginal effect of health improvement on agricultural employment was 2.11%, indicating that agricultural activities had a positive effect on maintaining the health of the elderly [32]. In addition, studies revealed that elder people’s participation in agricultural worked not only benefits their physical health, but also significantly improved their subjective well-being [33].

Overall, all groups showed a growth trend, but their growth base was lower than that of the RL group, and the overall growth rate in 2035 was as high as 69.07%, of which the growth rate of the TN group was 73.26%. The above results may be due to the improvement of the medical system and the remarkable improvement in the quality of medical services, which have led to a significant increase in the average life expectancy. However, this has also led to a surge in the number of elderly people, and if the related pension facilities cannot keep up with the population growth rate, the future pension industry will face a huge dilemma. In the future, the elderly will have the highest demand for outpatient services, reaching about 1.5 billion people by 2035. Family members and related staff can provide more help to the elderly in home care and community care models, and can provide better services by learning certain care knowledge. However, more in-depth physical diseases of the elderly require the participation of more professional doctors, and the replacement rate of doctors’ door-to-door services is very low, and the equipment they carry is also limited. Therefore, it is difficult for the above two types of care modes to meet the needs of the elderly. At the same time, the scale of meal assistance service can be further expanded, which can not only provide meals for the elderly but also enhance communication among the elderly and actively promote the physical health of the elderly.

The disability level prediction model constructed in this study has some universality, and the selected dimension is a common factor affecting the disability status of the elderly in the world. However, there are some differences in the cost structure of care in different countries. For example, institutional care is generally more expensive in developed countries, while institutional care may be a more economical option in some developing countries due to the lack of home care support. In addition, some countries share the cost of care through long-term care insurance, such as Japan and Germany. This system can effectively reduce the economic burden on families and society, but it needs to be adapted according to the country’s economic level and social security system. According to previous research, high-income countries tend to provide high-quality institutional care services, while low- and middle-income countries rely more on family and community care, which is consistent with the results of this study [34,35]. The comparative advantage of the elderly in rural China in terms of physical function is similar to the phenomenon of rural elderly in some developing countries (such as India and Brazil) maintaining higher physical functions due to their continuous engagement in agricultural labor. In urban areas, the increasing trend of chronic diseases and disability risks faced by elderly people in China is closer to the characteristics of the aging process in developed countries in Europe and America. This cross-regional comparison not only validates the explanatory power of the research model in different socio-economic contexts but also provides a classification reference for countries in formulating response strategies.

Based on the above assessment of disability level, prediction of disability scale, and analysis of nursing economic costs, corresponding improvement strategies can be proposed. Firstly, improvements should be made in the legal aspect to accelerate the legislative process of ECSs and bring the relevant systems for the care of DEP into the legal track. Concurrently, the three-level ECS network at the county, township, and village levels will be enhanced. A home-based community ECS circle will be established, and embedded ECS institutions will be developed within the community. The construction of a nursing system for DEP will be expedited. As a next step, there is a need to deepen the reform of the public elderly care institution system, emphasizing the expansion of the supply of inclusive, back-up and basic care services. This can improve the social support system for the care of persons with disabilities, strengthen visiting, and care services for older persons living alone, left behind and empty nesters, and raise the level of home-based care. Finally, improvements should be made in terms of talents, strengthening the construction of nursing teams, focusing on absorbing poor people and monitoring objects, preventing them from returning to poverty and employment, and guiding more willing and capable rural villagers or young and elderly people to participate in nursing positions. To ensure the feasibility of the above proposals, strict implementation will be carried out according to short-term and long-term implementation plans, and gradual progress will be made to strengthen interdepartmental coordination. Based on the projected number of DEP and their care needs, the government shall provide appropriate policy support and financial subsidies to encourage social forces to establish community ECS institutions. In addition, clear subsidy targets and standards should be set to enhance the flexibility and effectiveness of subsidies through various means, such as direct subsidies, service vouchers, and tax exemptions.

In summary, the study found that the community care model has significant advantages in terms of cost and satisfaction, and can provide more economical and humane services. Moreover, it may grow beyond the capacity of societies and families, especially as the population of disabled older people grows. Therefore, in the future, the study can focus on promoting the development of community-based ECSs, and share the economic burden of institutional care through policy instruments such as long-term care insurance, optimize the allocation of care resources, and improve the efficiency and quality of care services.

## 5 Conclusion

The experimental results showed that the physical fitness of the rural elderly was significantly better than that of the urban elderly, especially in the younger age group (60–79 years), the rural elderly had a lower level of disability, and the probability of entering the mild disability level was lower. This challenges traditional perceptions and suggested that policymakers needed to reassess the allocation of elderly care resources in urban and rural areas, taking into account the health advantages and potential needs of rural elderly. In addition, compared with the ME group, the probability of the HL group remaining healthy was as high as 77%, and the transfer probability of entering the mild disability level was as high as 67%. Meanwhile, there was a large difference between the TN group and the RL group, and the physical fitness of the elderly in the DL group in the RL group was significantly better than that in the TN group. The above results might be due to the fact that biologically, women have stronger antioxidant capacity and immune system function. Moreover, the estrogen level of women could protect the cardiovascular system to a certain extent and reduce the incidence of cardiovascular disease, while men were more susceptible to chronic diseases (such as cardiovascular disease, diabetes, etc.) at an advanced age. Furthermore, these conditions often lead to an increased risk of disability. The final projection showed that the cost of institutional care in the severe disability category could be 3.8 times that of home care by 2035, highlighting the relationship between population aging and the increase in care costs and the need for policy tools to share the economic burden of institutional care. In summary, the research method can accurately predict SoED, providing solid data support for the calculation of subsequent nursing costs and the proposal of improvement measures. This method is beneficial for better ensuring the life quality of the elderly, reducing the burden on families and society, and promoting social harmony and stability. However, this study still has certain limitations. For example, data such as chronic disease status rely on self-reporting by respondents, which may lead to recall bias or social desirability bias. Assessments of an older person’s own health status may be influenced by subjective perceptions, cultural background, or understanding of the disease, leading to biased assessments of disability levels. Future research can combine medical records, clinical examinations, or objective health monitoring device data to further improve the accuracy and reliability of assessments.

## Supporting information

S1 File Minimal Data Set. (DOC)
