# Supplementary material for: Prediction of disability levels and research on nursing economic costs for elderly people in China
Source: PLoS One. 2025 Nov 17;20(11):e0336605. doi: 10.1371/journal.pone.0336605 (PMC12622809; doi:10.1371/journal.pone.0336605)
Supplement: S1 File — (DOC) [file pone.0336605.s001.doc]

**The data in Figure 4**

| Group | Category | DL group | | | | HL group | | | |
| --- | --- | --- | --- | --- | --- | --- | --- | --- | --- |
|  |  | Healthy | Mild disability | Moderate disability | Severe disability | Healthy | Mild disability | Moderate disability | Severe disability |
| ME | Healthy | 0.3301 | 0.5885 | 0.0766 | 0.0048 | 0.2222 | 0.6667 | 0.1111 | 0.0000 |
|  | Mild disability | 0.0661 | 0.6748 | 0.2382 | 0.0208 | 0.0364 | 0.5455 | 0.3727 | 0.0455 |
|  | Moderate disability | 0.0270 | 0.4369 | 0.4595 | 0.0766 | 0.0000 | 0.2647 | 0.5294 | 0.2059 |
|  | Severe disability | 0.0000 | 0.0000 | 0.4000 | 0.6000 | 0.0000 | 0.0000 | 0.6667 | 0.3333 |
| FE group | Healthy | 0.3525 | 0.5656 | 0.0820 | 0.0000 | 0.7500 | 0.2500 | 0.0000 | 0.0000 |
|  | Mild disability | 0.0503 | 0.6778 | 0.2448 | 0.0271 | 0.0638 | 0.5319 | 0.3830 | 0.0213 |
|  | Moderate disability | 0.0154 | 0.3641 | 0.5179 | 0.1026 | 0.0385 | 0.2692 | 0.4615 | 0.2308 |
|  | Severe disability | 0.0000 | 0.1875 | 0.0625 | 0.7500 | 0.0000 | 0.0000 | 1.0000 | 0.0000 |

**The data in Figure 5**

| Project | Category | EL group | | | | DL group | | | |
| --- | --- | --- | --- | --- | --- | --- | --- | --- | --- |
|  |  | Healthy | Mild disability | Moderate disability | Severe disability | Healthy | Mild disability | Moderate disability | Severe disability |
| Probability matrix of disability level transition between TN group and age group | Healthy | 0.2500 | 0.5000 | 0.2500 | 0.0000 | 0.2041 | 0.6939 | 0.0952 | 0.0068 |
|  | Mild disability | 0.0435 | 0.4928 | 0.4203 | 0.0435 | 0.0423 | 0.6382 | 0.2896 | 0.0299 |
|  | Moderate disability | 0.0000 | 0.2500 | 0.5357 | 0.2143 | 0.0228 | 0.3648 | 0.5179 | 0.0945 |
|  | Severe disability | 0.0000 | 0.0000 | 0.7500 | 0.2500 | 0.0000 | 0.1429 | 0.0000 | 0.8571 |
| Probability matrix of disability level transition between RL group and age group | Healthy | 0.4444 | 0.5556 | 0.0000 | 0.0000 | 0.4457 | 0.4891 | 0.0652 | 0.0000 |
|  | Mild disability | 0.0455 | 0.5795 | 0.3409 | 0.0341 | 0.0860 | 0.7339 | 0.1667 | 0.0134 |
|  | Moderate disability | 0.0313 | 0.2813 | 0.4688 | 0.2188 | 0.0182 | 0.5091 | 0.4000 | 0.0727 |
|  | Severe disability | 0.0000 | 0.0000 | 0.0000 | 1.0000 | 0.0000 | 0.1429 | 0.4286 | 0.4286 |

**The data in Figure 6**

| Project | Group | | DL group | | | | HL group | | | |
| --- | --- | --- | --- | --- | --- | --- | --- | --- | --- | --- |
|  |  |  | Healthy | Mild disability | Moderate disability | Severe disability | Healthy | Mild disability | Moderate disability | Severe disability |
| Transition probability/% | ME | Healthy | 0.7685 | 0.2001 | 0.0298 | 0.0016 | 0.7357 | 0.2222 | 0.0370 | 0.0050 |
|  |  | Mild disability | 0.0220 | 0.8720 | 0.0990 | 0.0069 | 0.0121 | 0.8087 | 0.1640 | 0.0152 |
|  |  | Moderate disability | 0.0090 | 0.1456 | 0.8198 | 0.0255 | 0.0000 | 0.0882 | 0.8431 | 0.0686 |
|  |  | Severe disability | 0.0000 | 0.0000 | 0.1333 | 0.8667 | 0.0000 | 0.0000 | 0.2222 | 0.7778 |
|  | FE | Healthy | 0.7736 | 0.1885 | 0.0379 | 0.0000 | 0.9167 | 0.0833 | 0.0000 | 0.0000 |
|  |  | Mild disability | 0.0161 | 0.8833 | 0.0916 | 0.0090 | 0.0213 | 0.8304 | 0.1277 | 0.0207 |
|  |  | Moderate disability | 0.0050 | 0.1214 | 0.8394 | 0.0342 | 0.0228 | 0.0897 | 0.8006 | 0.0869 |
|  |  | Severe disability | 0.0000 | 0.0325 | 0.0508 | 0.9167 | 0.0000 | 0.0000 | 0.3455 | 0.6545 |
|  | TN | Healthy | 0.6841 | 0.2219 | 0.0917 | 0.0023 | 0.7500 | 0.1667 | 0.0833 | 0.0000 |
|  |  | Mild disability | 0.0141 | 0.8751 | 0.0965 | 0.0143 | 0.0184 | 0.7815 | 0.1856 | 0.0145 |
|  |  | Moderate disability | 0.0076 | 0.1216 | 0.8393 | 0.0315 | 0.0000 | 0.0833 | 0.8452 | 0.0714 |
|  |  | Severe disability | 0.0000 | 0.0476 | 0.1667 | 0.7857 | 0.0000 | 0.0000 | 0.2500 | 0.7500 |
|  | RL | Healthy | 0.8041 | 0.1630 | 0.0317 | 0.0012 | 0.8221 | 0.1759 | 0.0015 | 0.0005 |
|  |  | Mild disability | 0.0287 | 0.9090 | 0.0578 | 0.0045 | 0.0152 | 0.8399 | 0.1336 | 0.0114 |
|  |  | Moderate disability | 0.0061 | 0.1616 | 0.8081 | 0.0242 | 0.0104 | 0.0938 | 0.8229 | 0.0729 |
|  |  | Severe disability | 0.0000 | 0.0476 | 0.1429 | 0.8095 | 0.0000 | 0.0000 | 0.1006 | 0.8994 |
| Correction error/% | ME | | -4.6530 | 2.0988 | -3.8115 | 2.7225 | -2.2671 | 2.7720 | -3.5244 | 1.8612 |
|  | FE | | -2.5542 | 0.0198 | 0.7029 | -0.1188 | -3.4749 | -3.6333 | 4.7223 | -0.2772 |
|  | TN | | -0.5049 | -1.9107 | 3.0987 | 3.6927 | -1.9008 | 1.8612 | -1.7424 | 1.0890 |
|  | RL | | -0.3762 | 0.6237 | 2.4255 | 1.9899 | 0.5742 | 0.3861 | 0.2079 | 1.0494 |

**The data in Figure 7**

| Year | ME Group | FE Group | TN Group | RL Group | Total population |
| --- | --- | --- | --- | --- | --- |
| 2025 | 13.82 | 12.62 | 11.72 | 10.72 | 26.44 |
| 2026 | 14.27 | 13.09 | 12.22 | 11.04 | 27.36 |
| 2027 | 14.73 | 13.57 | 12.74 | 11.16 | 28.30 |
| 2028 | 15.21 | 14.07 | 13.28 | 11.23 | 29.28 |
| 2029 | 15.70 | 14.59 | 13.84 | 11.35 | 30.29 |
| 2030 | 16.21 | 15.13 | 14.42 | 11.52 | 31.34 |
| 2031 | 16.74 | 15.69 | 15.02 | 11.71 | 32.43 |
| 2032 | 17.29 | 16.27 | 15.65 | 11.91 | 33.56 |
| 2033 | 17.86 | 16.87 | 16.30 | 12.13 | 34.73 |
| 2034 | 18.45 | 16.98 | 16.67 | 12.37 | 35.43 |
| 2035 | 20.95 | 17.18 | 16.78 | 12.45 | 38.13 |

**The data in Figure 8**

| Year | ME Group | FE Group | TN Group | RL Group | Total population |
| --- | --- | --- | --- | --- | --- |
| 2025 | 2.76 | 2.20 | 1.95 | 1.87 | 4.96 |
| 2026 | 2.84 | 2.28 | 2.03 | 1.92 | 5.12 |
| 2027 | 2.79 | 2.36 | 2.11 | 1.94 | 5.15 |
| 2028 | 2.80 | 2.45 | 2.20 | 1.95 | 5.25 |
| 2029 | 2.82 | 2.54 | 2.30 | 1.97 | 5.36 |
| 2030 | 2.86 | 2.63 | 2.39 | 2.00 | 5.49 |
| 2031 | 2.97 | 2.73 | 2.49 | 2.04 | 5.70 |
| 2032 | 3.01 | 2.83 | 2.60 | 2.07 | 5.84 |
| 2033 | 3.05 | 2.94 | 2.71 | 2.11 | 5.99 |
| 2034 | 3.17 | 2.95 | 2.77 | 2.15 | 6.12 |
| 2035 | 3.29 | 3.02 | 2.79 | 2.17 | 6.31 |

**The data in Figure 9**

| Project | Year | ME Group - healthy | ME Group - mild disability | ME Group - moderate disability | ME Group - severe disability | FE Group - healthy | FE Group - mild disability | FE Group - moderate disability | FE Group - severe disability |
| --- | --- | --- | --- | --- | --- | --- | --- | --- | --- |
| DL Group | 2025 | 0.3038 | 0.4966 | 0.0329 | 0.0360 | 0.2480 | 0.4957 | 0.0030 | 0.0563 |
|  | 2026 | 0.3119 | 0.4908 | 0.0333 | 0.0372 | 0.2530 | 0.4908 | 0.0030 | 0.0555 |
|  | 2027 | 0.3200 | 0.4850 | 0.0337 | 0.0384 | 0.2570 | 0.4860 | 0.0030 | 0.0548 |
|  | 2028 | 0.3251 | 0.4792 | 0.0340 | 0.0396 | 0.2630 | 0.4811 | 0.0030 | 0.0540 |
|  | 2029 | 0.3362 | 0.4734 | 0.0344 | 0.0408 | 0.2660 | 0.4763 | 0.0031 | 0.0533 |
|  | 2030 | 0.3393 | 0.4675 | 0.0347 | 0.0420 | 0.2720 | 0.4714 | 0.0031 | 0.0525 |
|  | 2031 | 0.3424 | 0.4617 | 0.0351 | 0.0432 | 0.2790 | 0.4666 | 0.0031 | 0.0518 |
|  | 2032 | 0.3305 | 0.4559 | 0.0355 | 0.0444 | 0.2860 | 0.4617 | 0.0031 | 0.0510 |
|  | 2033 | 0.3386 | 0.4501 | 0.0358 | 0.0456 | 0.2890 | 0.4569 | 0.0031 | 0.0503 |
|  | 2034 | 0.3313 | 0.4443 | 0.0362 | 0.0468 | 0.2910 | 0.4520 | 0.0032 | 0.0495 |
|  | 2035 | 0.3378 | 0.4384 | 0.0365 | 0.0480 | 0.2980 | 0.4472 | 0.0032 | 0.0488 |
| HL Group | 2025 | 0.0428 | 0.5650 | 0.3553 | 0.1520 | 0.0268 | 0.5071 | 0.4350 | 0.1162 |
|  | 2026 | 0.0419 | 0.5725 | 0.3518 | 0.1582 | 0.0264 | 0.5126 | 0.4292 | 0.1169 |
|  | 2027 | 0.0410 | 0.5800 | 0.3483 | 0.1544 | 0.0259 | 0.5181 | 0.4234 | 0.1176 |
|  | 2028 | 0.0401 | 0.5875 | 0.3448 | 0.1506 | 0.0255 | 0.5236 | 0.4176 | 0.1183 |
|  | 2029 | 0.0392 | 0.5950 | 0.3413 | 0.1568 | 0.0250 | 0.5291 | 0.4118 | 0.1190 |
|  | 2030 | 0.0383 | 0.5880 | 0.3378 | 0.1430 | 0.0246 | 0.5346 | 0.4060 | 0.1197 |
|  | 2031 | 0.0374 | 0.5954 | 0.3343 | 0.1492 | 0.0241 | 0.5401 | 0.4002 | 0.1204 |
|  | 2032 | 0.0365 | 0.5829 | 0.3308 | 0.1454 | 0.0137 | 0.5456 | 0.3944 | 0.1211 |
|  | 2033 | 0.0356 | 0.5900 | 0.3273 | 0.1416 | 0.1232 | 0.5511 | 0.3886 | 0.1218 |
|  | 2034 | 0.0347 | 0.5971 | 0.3238 | 0.1478 | 0.0128 | 0.5566 | 0.3828 | 0.1225 |
|  | 2035 | 0.0338 | 0.5994 | 0.3203 | 0.1410 | 0.0123 | 0.5621 | 0.3770 | 0.1232 |

**The data in Figure 10**

| Project | Year | ME Group - healthy | ME Group - mild disability | ME Group - moderate disability | ME Group - severe disability | FE Group - healthy | FE Group - mild disability | FE Group - moderate disability | FE Group - severe disability |
| --- | --- | --- | --- | --- | --- | --- | --- | --- | --- |
| Comparison of results by gender | 2025 | 0.348 | 9.888 | 5.733 | 0.423 | 0.826 | 9.756 | 6.138 | 0.127 |
|  | 2026 | 0.570 | 10.222 | 6.006 | 0.434 | 0.918 | 10.117 | 7.099 | 0.170 |
|  | 2027 | 1.658 | 10.565 | 6.342 | 0.445 | 1.009 | 10.889 | 7.386 | 1.217 |
|  | 2028 | 2.747 | 10.918 | 6.657 | 0.456 | 2.100 | 11.271 | 7.673 | 1.264 |
|  | 2029 | 3.865 | 11.272 | 6.993 | 0.467 | 3.192 | 11.663 | 7.961 | 1.312 |
|  | 2030 | 3.953 | 11.636 | 7.329 | 0.478 | 4.283 | 12.055 | 8.248 | 1.359 |
|  | 2031 | 4.042 | 11.999 | 7.565 | 0.489 | 4.374 | 12.457 | 8.535 | 1.406 |
|  | 2032 | 4.160 | 12.373 | 7.544 | 0.501 | 4.465 | 12.859 | 8.822 | 1.453 |
|  | 2033 | 4.248 | 12.747 | 7.861 | 0.512 | 4.557 | 13.271 | 9.109 | 1.501 |
|  | 2034 | 4.337 | 13.130 | 8.177 | 0.523 | 4.648 | 13.683 | 9.396 | 1.548 |
|  | 2035 | 4.455 | 14.968 | 8.494 | 0.534 | 4.739 | 14.985 | 9.683 | 1.595 |
| Comparison of urban and rural results | Year | TN Group - healthy | TN Group - mild disability | TN Group - moderate disability | TN Group - severe disability | RL Group - healthy | RL Group - mild disability | RL Group - moderate disability | RL Group - severe disability |
|  | 2025 | 0.994 | 7.030 | 2.060 | 0.160 | 0.379 | 7.092 | 3.976 | 0.116 |
|  | 2026 | 1.019 | 7.310 | 2.158 | 0.164 | 0.404 | 7.340 | 4.092 | 0.121 |
|  | 2027 | 1.043 | 7.590 | 2.257 | 0.168 | 0.629 | 7.587 | 4.207 | 0.127 |
|  | 2028 | 1.067 | 7.870 | 2.356 | 0.172 | 0.755 | 7.835 | 4.323 | 0.132 |
|  | 2029 | 1.091 | 8.150 | 2.455 | 0.176 | 0.980 | 8.083 | 5.439 | 0.138 |
|  | 2030 | 1.115 | 8.430 | 2.554 | 0.179 | 1.005 | 8.331 | 5.555 | 0.143 |
|  | 2031 | 1.140 | 8.710 | 2.652 | 0.183 | 1.030 | 8.579 | 6.292 | 0.149 |
|  | 2032 | 1.164 | 8.990 | 2.751 | 0.187 | 1.056 | 8.826 | 6.448 | 0.154 |
|  | 2033 | 1.188 | 9.270 | 2.850 | 0.191 | 1.081 | 9.074 | 6.604 | 0.160 |
|  | 2034 | 1.212 | 9.550 | 2.949 | 0.195 | 1.106 | 9.322 | 6.760 | 0.165 |
|  | 2035 | 1.236 | 9.630 | 3.048 | 0.199 | 1.132 | 9.346 | 6.916 | 0.171 |

**The data in Figure 10**

| Project | Year | ME Group - healthy | ME Group - mild disability | ME Group - moderate disability | ME Group - severe disability | FE Group - healthy | FE Group - mild disability | FE Group - moderate disability | FE Group - severe disability |
| --- | --- | --- | --- | --- | --- | --- | --- | --- | --- |
| Comparison of results by gender | 2025 | 0.348 | 9.888 | 0.988 | 0.368 | 0.339 | 9.756 | 18.138 | 0.052 |
|  | 2026 | 0.570 | 10.222 | 1.225 | 1.378 | 0.376 | 10.017 | 19.099 | 2.070 |
|  | 2027 | 0.763 | 10.565 | 2.518 | 2.387 | 0.414 | 10.189 | 20.386 | 4.499 |
|  | 2028 | 1.264 | 10.918 | 3.792 | 3.397 | 0.861 | 10.271 | 21.673 | 6.035 |
|  | 2029 | 1.778 | 11.272 | 4.084 | 4.076 | 1.309 | 10.663 | 22.961 | 8.454 |
|  | 2030 | 1.818 | 11.636 | 4.376 | 6.384 | 1.756 | 11.055 | 24.248 | 11.864 |
|  | 2031 | 1.859 | 11.999 | 5.582 | 8.692 | 1.793 | 11.457 | 25.535 | 12.274 |
|  | 2032 | 1.914 | 12.373 | 6.563 | 10.028 | 1.831 | 11.859 | 26.822 | 12.685 |
|  | 2033 | 1.954 | 12.747 | 6.839 | 12.336 | 1.868 | 12.271 | 28.109 | 13.104 |
|  | 2034 | 1.995 | 13.130 | 7.114 | 13.644 | 1.906 | 13.083 | 29.396 | 13.514 |
|  | 2035 | 2.049 | 14.968 | 7.390 | 14.952 | 1.943 | 14.953 | 29.683 | 13.924 |
| Comparison of urban and rural results | Year | TN Group - healthy | TN Group - mild disability | TN Group - moderate disability | TN Group - severe disability | RL Group - healthy | RL Group - mild disability | RL Group - moderate disability | RL Group - severe disability |
|  | 2025 | 0.099 | 6.116 | 8.118 | 2.774 | 0.179 | 6.312 | 5.239 | 2.289 |
|  | 2026 | 0.102 | 6.360 | 10.321 | 2.911 | 0.204 | 6.533 | 5.422 | 2.342 |
|  | 2027 | 0.104 | 6.603 | 12.524 | 3.049 | 0.629 | 6.752 | 5.605 | 2.395 |
|  | 2028 | 0.107 | 6.747 | 14.727 | 3.187 | 0.755 | 6.973 | 5.788 | 2.449 |
|  | 2029 | 0.109 | 6.631 | 16.930 | 3.325 | 0.980 | 7.089 | 5.884 | 2.962 |
|  | 2030 | 0.112 | 6.660 | 18.032 | 3.462 | 1.005 | 7.091 | 5.886 | 3.015 |
|  | 2031 | 0.114 | 6.881 | 18.435 | 3.600 | 1.030 | 7.121 | 5.910 | 3.354 |
|  | 2032 | 0.116 | 7.102 | 18.838 | 3.738 | 1.056 | 7.326 | 6.080 | 4.767 |
|  | 2033 | 0.119 | 7.323 | 19.241 | 3.876 | 1.081 | 7.531 | 6.251 | 4.867 |
|  | 2034 | 0.121 | 7.545 | 19.644 | 4.013 | 1.106 | 7.737 | 6.422 | 4.966 |
|  | 2035 | 0.124 | 7.608 | 20.047 | 4.496 | 1.132 | 7.757 | 6.438 | 5.066 |

**The data in Figure 12**

| Year | Home medical service | Home service for household chores | Rehabilitation nursing services | Psychological counseling or chat to relieve boredom services | Health education services | Daytime care services | Bathing assistance service | Meal assistance service | Meal assistance service |
| --- | --- | --- | --- | --- | --- | --- | --- | --- | --- |
| 2025 | 10.03 | 3.37 | 2.07 | 1.50 | 2.11 | 2.14 | 1.11 | 2.03 | 1.15 |
| 2026 | 10.67 | 3.57 | 2.11 | 1.70 | 2.30 | 2.20 | 1.30 | 2.07 | 1.23 |
| 2027 | 11.02 | 3.62 | 2.34 | 1.80 | 2.40 | 2.30 | 1.40 | 2.12 | 1.36 |
| 2028 | 11.57 | 3.85 | 2.58 | 1.90 | 2.50 | 2.50 | 1.50 | 2.14 | 1.48 |
| 2029 | 11.93 | 3.96 | 2.76 | 2.10 | 2.70 | 2.60 | 1.60 | 2.26 | 1.59 |
| 2030 | 12.65 | 4.01 | 2.84 | 2.30 | 2.90 | 2.70 | 1.70 | 2.57 | 1.60 |
| 2031 | 12.97 | 4.25 | 2.95 | 2.70 | 3.00 | 2.80 | 1.74 | 2.94 | 1.62 |
| 2032 | 13.47 | 4.36 | 3.06 | 2.90 | 3.10 | 2.90 | 1.76 | 3.02 | 1.65 |
| 2033 | 14.02 | 4.75 | 3.24 | 3.20 | 3.20 | 3.20 | 1.79 | 3.27 | 1.72 |
| 2034 | 15.13 | 5.02 | 3.58 | 3.50 | 3.50 | 3.50 | 1.82 | 3.58 | 1.85 |
| 2035 | 15.36 | 5.23 | 3.79 | 3.60 | 3.76 | 3.71 | 1.84 | 3.85 | 1.87 |

**The data in Figure 13**

| Year | Number of disabled persons/billion people | | | Nursing cost/10^7^ yuan | | |
| --- | --- | --- | --- | --- | --- | --- |
|  | Mild | Moderate | Severe | Mild | Moderate | Severe |
| 2025 | 14.117 | 7.938 | 2.339 | 5.174 | 3.772 | 2.318 |
| 2026 | 13.851 | 8.210 | 2.772 | 5.076 | 3.917 | 2.747 |
| 2027 | 14.151 | 9.130 | 3.190 | 5.186 | 4.136 | 3.161 |
| 2028 | 14.853 | 9.699 | 3.617 | 5.443 | 4.428 | 3.584 |
| 2029 | 15.412 | 10.519 | 3.949 | 5.648 | 4.651 | 3.913 |
| 2030 | 16.013 | 11.013 | 4.244 | 5.868 | 4.869 | 4.205 |
| 2031 | 16.639 | 11.501 | 4.510 | 6.098 | 5.085 | 4.470 |
| 2032 | 17.135 | 11.882 | 4.716 | 6.280 | 5.253 | 4.673 |
| 2033 | 17.863 | 12.414 | 4.967 | 6.546 | 5.488 | 4.922 |
| 2034 | 18.497 | 12.872 | 5.178 | 6.779 | 5.691 | 5.132 |
| 2035 | 19.187 | 13.366 | 5.397 | 7.032 | 5.909 | 5.348 |
